# Acclimation of sugar beet in morphological, physiological and *BvAMT1*.*2* expression under low and high nitrogen supply

**DOI:** 10.1371/journal.pone.0278327

**Published:** 2022-11-29

**Authors:** Jiajia Li, Wangsheng Li, Lingqing Xu, Man Wang, Wanting Zhou, Siqi Li, Wenbo Tan, Qiuhong Wang, Wang Xing, Dali Liu

**Affiliations:** 1 National Beet Medium-Term Gene Bank, Heilongjiang University, Harbin, P. R. China; 2 Key Laboratory of Sugar Beet Genetics and Breeding, Heilongjiang Province Common College/College of Advanced Agriculture and Ecological Environment, Heilongjiang University, Harbin, P. R. China; National Taiwan University, TAIWAN

## Abstract

Understanding the response and tolerance mechanisms of nitrogen (N) stress is essential for the taproot plant of sugar beet. Hence, in this study, low (0.5 and 3 mmol/L; N0.5 and N3), moderate (5 mmol/L; N5; control) and high (10 and 12 mmol/L; N10 and N12) N were imposed to sugar beet to comparatively investigate the growth and physiological changes, and expression pattern of the gene involving ammonia transporting at different seedling stages. The results showed that, different from N5 which could induce maximum biomass of beet seedlings, low N was more likely to inhibit the growth of beet seedlings than high N treatments. Morphological differences and adverse factors increased significantly with extension of stress time, but sugar beet seedlings displayed a variety of physical responses to different N concentrations to adapt to N abnormal. At 14 d, the chlorophyll content, leaf and root surface area, total dry weight and nitrogen content of seedlings treated with N0.5 decreased 15.83%, 53.65%, 73.94%, 78.08% and 24.88% respectively, compared with N12; however, the root shoot ratio increased significantly as well as superoxide dismutase (SOD), peroxidase (POD), glutamine synthetase (GS) activity and malondialdehyde (MDA) and proline content, especially in root. The expression of *BvAMT1*.*2* was also regulated in an N concentration-dependent manner, and was mainly involved in the tolerance of beet leaves to N stress, which significantly positively correlated to GS activity on the basis of its high affinity to N. It can be deduced that the stored nutrients under low N could only maintain relatively stable root growth, and faced difficulty in being transported to the shoots. Sugar beet was relatively resilient to N0.5 stress according to the mean affiliation function analysis. These results provide a theoretical basis for the extensive cultivation of sugar beet in N-stressed soil.

## 1. Introduction

In the last decade, the N fertilizer utilization rates of rice (*Oryza sativa*), wheat (*Triticum aestivum*), and maize (*Zea mays*) in China have been 39.0%, 34.8%, and 29.1%, respectively [1], which are far below international levels. China’s N fertilizer consumption is only 26% of the world’s consumption to produce 21% of the world’s grain [2]. Higher N application can lead to lower N use efficiency [3] and excessive N input can lead to increased nitrate leaching and environmental pollution [4]. On the contrary, studies have shown that insufficient nutrient availability can seriously affect plant growth [5]. Understanding the response of plants to low and high N stress is crucial for effective agricultural fertilization and selection of environmental resistant varieties.

N is a decisive factor for high yields in agriculture [6]. N is an important component of chlorophyll [7], and it affects photosynthesis and growth rate of plants through influencing N and chlorophyll content and total biomass [8]. Thus, reduction in the photosynthetic capacity of plants induced by N deficiency is one of the most important reasons for inhibited plant growth and development. Roots are not only an important organ for nutrient and water uptake, but are also the first organ to sense adversity signals from soil stressors [9]. Mild N deficiency promotes root elongation and facilitates deep root penetration, while appropriate N application increases root number, biomass, and density; however, excessive N application inhibits root growth [10]. Within a certain range, root biomass and oxidative activity in root increase with increasing N levels [11]. Under adverse stress, plants initiate adaptive changes in physiology and morphology, which affect internal metabolic processes, to maintain survival [12].

Ammonium is a compound of emerging interest because increased CO_2_ environments are expected to reduce the use of nitrate by C_3_ species [13]. There are two sources of ammonium in plants, one being nitrate and the other that is taken up directly from the soil via ammonia transporters (AMTs) and assimilated as amino acids via the GS/GOGAT cycle [12]. Glutamine synthetase (GS) is the key enzyme for ammonia assimilation [14] and is responsible for regulating plant nitrogen reuse, which is known to be closely related to seed germination, leaf senescence, and nitrogen accumulation processes [15]. Increased GS activity facilitates plant ammonia assimilation and N translocation, and as a result, NH_4_^+^ levels in plant remain consistently low; thus, preventing the toxicity caused by excessive ammonium [16, 17]. The pineapple *GS1* gene was introduced into young aspen trees, resulting in improved N assimilation efficiency and growth promotion [18]. With respect to the studies on plants *AMT*s [19, 20], it was observed that, *AMT1*.*2* had a different regulatory pattern that could respond to both NH_4_^+^ or NO_3_^-^ [19], and mainly functioned in the endodermis, mediating ATP that facilitates the partitioning of NH_4_^+^ to shoots [21]. Besides, overexpression of *BcAMT1*.*2* may affect the homeostasis between nitrogen and carbon, thus regulating plant growth [19].

When plants are subjected to stress, the balance between the production and elimination of intercellular reactive oxygen species (ROS) is disrupted, and evolved complex corresponding defense systems [22]. The primary antioxidant enzymes that scavenge ROS in plant cells are peroxidase (POD) and superoxide dismutase (SOD), and the activity of antioxidant enzyme is related to the ability of plants to adapt to adverse stress [23]. One of the products of membrane lipid peroxidation is malondialdehyde (MDA), whose content reflects the degree of membrane damage in plants [24]. Proline is an important osmoregulatory substance and effective hydroxyl radical scavenger in organisms, and can effectively protect plants from stressful conditions [25]. Numerous studies have revealed that N stress impacts plant normal physical metabolic activities, thus eventually decrease biomass accumulation [26]. Plants can ameliorate the damages of N stress by enhancing soluble protein content, and promoting N assimilation [27].

Sugar beet (*Beta vulgaris* L.) is a biennial heterogeneous pollinated crop, which is important in sugar production and non- food energy supply [28, 29]. Nitrogen is the most important nutrient limiting factor to sugar beet, which growth and taproot development directly related to N level in surrounding environment [30]. It was found that N deficiency led to low yields, but too much N reduced root quality in sugar beet [31, 32]. Presently, there are few records on the response and potential adaptation mechanisms of sugar beet seedlings under reduced and high N environment.

Therefore, in this study, we focused on the growth index, antioxidant enzymes, MDA, as well as proline and functional gene expression under short- and long-term N treatments to determine the intervals of N tolerance and N metabolite response in sugar beet seedlings. The objectives were: (1) to compare the processes of morphological, physical and *AMT* gene expression modifications of beet seedlings under different N treatment; (2) to determine the correlation between the above factors, and (3) to elucidate the adaptation mechanism of sugar beet to N deficiency or excessive N. The results are useful for the rational use of N fertilizer to achieve N reduction and increased efficiency.

## 2. Materials and methods

### 2.1 Experimental materials and culture conditions

Sugar beet ‘92021-1-1’ germplasm from the National Beet Medium-term Gene Bank of Heilongjiang University, which not only had a high germination rate, but also germinated rapidly, emerged clearly, and had robust and vigorous seedlings, was selected for the following nitrogen stress treatments.

Seeds of uniform size and free from pests and diseases were soaked overnight in 2‰ thiram solution and rinsed several times with distilled water. Subsequently, they were sowed in seedling trays containing vermiculite. After 8 d of incubation, the seedlings were transplanted into 0 mmol/L (N deficiency), 0.5 and 3 mmol/L (low N), 5 mmol/L (moderate N), and 10 and 12 mmol/L (high N) modified Hoagland nutrient solutions (S1 Table). Each treatment was conducted in triplicates. Samples were collected after 7, 14, and 20 d of N treatment at 9 A.M, and stored at -80°C after being snap-frozen in liquid N. The hydroponic conditions were 25°C during the day and 18°C at night with a 14 h light/10 h dark cycle and 200 μmol/(m^2^•s) light intensity.

### 2.2 Measurement of morphological index of sugar beet

The sugar beet seedlings under different N stresses were observed, and the plant height, root length, and fresh weight were measured carefully. Images of the root system and leaves were obtained using Epson Expression 1680 Scanner (Seiko Epson Corp., Tokyo, Japan) at a resolution of 800 dpi. Morphological index, such as total root surface area, number of root bifurcations, and leaf area were analyzed using the Win Rhizo analysis system. The above-ground and roots were oven-dried at 105°C for 30 min and further dried at 75°C to constant weight. The dry weights were measured and recorded.

### 2.3 Chlorophyll and total nitrogen content determination

Samples weighing 0.5 g were ground to powder with liquid nitrogen. Ten milliliters of 95% ethanol was added to extract chlorophyll by centrifugation at 4°C and 3000 rpm for 15 min. The amount of chlorophyll a (λ = 665) and chlorophyll b (λ = 649) in the samples was determined using a spectrophotometer (UV-1800 240 V, Shimadzu Corporation, Kyoto, Japan) and the amount of chlorophyll was calculated according to the following formula [33].


$$C_{a}(mg|L)=13.95*A_{665}-6.88*A_{649}$$

$$C_{b}(mg|L)=24.96*A_{649}-7.32*A_{665}$$

chloroplastpigmentcontent(mg|g)=((C*V*N)|(m*1000))

$$C_{a+b}\;(mg/g)=C_{a}+C_{b}$$

Where C is the pigment content (mg/L); V is the volume of the extract (ml); N is the dilution multiple; m is the sample mass (g); 1000 indicated 1L = 1000 ml.

The leaves and roots were rinsed with deionized water, oven-dried at 105°C for 30 min and further dried at 65°C until to constant weight. The dried samples (0.1 g) were crushed and ground, and were digested with concentrated sulfuric acid, catalyzing with potassium sulfate and copper sulfate, and. The N content was determined using a Hanon K1100 automatic kjeldahl nitrogen analyzer.

### 2.4 Antioxidant enzymes and glutamine synthetase activities assay

Superoxide dismutase (SOD) activity was determined using the nitrogen blue tetrazolium method (NBT): briefly, 0.5 g of sample was ground using pre-cooled phosphate buffer (pH = 7.8) and centrifuged at 4000 rpm for 10 min. The supernatant was taken as the SOD crude enzyme solution, using no light as a blank, and the absorbance value was measured at 560 nm on an enzyme calibrator [33].

For peroxidase (POD) activity assay, the reaction solution for determination was prepared by adding 28 μl of 0.2% guaiacol into 50 ml of 0.1 PBS (pH 7.0), heating (37°C) and stirring thoroughly; subsequently, the solution was cooled quickly, and 19 μl of 30% H_2_O_2_ was added. The dynamic absorbance at 470 nm was recorded using a UV spectrophotometer [33].

GS activity was measured according to instruction of Nanjing Institute of Built Bioengineering Kit (product no. A047-1-1). The supernatant was obtained after centrifugation at 8000 *g* for 10 min at 4°C and placed on ice for measurement. Enzyme activity was defined as one unit of enzyme activity per unit mass of sample per milliliter of reaction system, resulting in a change in absorbance value at 540 nm of 0.01 per minute.

### 2.5 Determination of lipid peroxidation

Lipid peroxidation was analyzed by measuring the content of malondialdehyde (MDA) in leaves and roots of sugar beet seedlings. MDA content was determined by the thiobarbituric acid (TCA) method. Sample of 0.5 g was ground with 5% TCA to obtain a homogenate, which was centrifuged at 3000 rpm for 10 min. Thereafter, 2 ml of supernatant was mixed with 2 ml of 0.67% TBA, and boiled for 30 min at 100°C. MDA was measured using a spectrophotometer (UV-1800 240 V, Shimadzu Corporation, Kyoto, Japan) to determine the absorbance values at wavelengths of 450 nm, 532 nm, 600 nm [33].

### 2.6 Measurement of protein and proline content

Protein content was determined using a biosharp BCA protein concentration kit (product no. BL521A) and the absorbance value was measured at 562 nm. The sample without BSA was used as a blank. The standard curve using A562 as the vertical coordinate and BSA content as the horizontal coordinate was used to calculate the protein concentration.

The proline content was determined by the acidic ninhydrin method. 0.5 g sample was ground with 3% sulfosalicylic acid, to which, added 2 ml of glacial acetic acid and ninhydrin. The mixture was boiled for 30 min at 100°C and extracted with 4 ml of toluene. The upper layer of red toluene solution was gently aspirated with a pipette, and the absorbance values were recorded using a colorimetry at 520 nm on a spectrophotometer (UV-1800 240 V, Shimadzu Corporation, Kyoto, Japan) using toluene as the blank [33].

### 2.7 Transcriptional expression of *BvAMT1*.*2* gene

RNA was extracted from sugar beet leaves and roots using the Vazyme #R701-01 kit (Nanjing Novozymes Biotech Co), and the TransScript One-Step gDNA Removal and cDNA Synthesis SuperMix Reverse Transcription Kit (Beijing All Style Gold Biotechnology Co) was used for the cDNA first strand synthesis. *BvGAPDH* (NC_024800) was selected as internal reference. To validate the expression of *BvAMT1*.*2* gene, quantitative real-time PCR (qRT-PCR) was performed using SuperReal PreMix Plus kit (TianGen) with three replicates for each sample. The reaction system (20 μl) was: 2×SuperReal PreMix Plus 10 μl, Primer-F 0.6 μl, Primer-R 0.6 μl, and diluted cDNA 1μl. PCR procedure was: pre-denaturation at 95°C for 15 min; denaturation at 95°C for 10 s, annealing at 60°C for 20 s, extension at 72°C for 32 s, 40 cycles; melting curve at 65–95°C. Primers used for qRT-PCR are shown in Table 1. Statistical analysis of gene expression was performed using the 2^−ΔΔ*CT*^ method.

**Table 1:** qRT-PCR primer sequences.

Gene	Location		Primer sequence (5’-3’)
*BvAMT1*.*2*	NC_025815.2	Forward	CGACTACATTGGCTGGAT
		Reverse	CAAGGCTCAACTACTGAACAC
*BvGAPDH*	NC_024800	Forward	GCTTTGAACGACCACTTCGC
		Reverse	ACGCCGAGAGCAACTTGAAC

### 2.8 Statistical analysis

The fuzzy mathematical membership function method was used to comprehensively evaluate the adaptation of sugar beet seedlings to N stress. There were "n" N stress levels (n = 1,…, i) and "m" indexes (m = 1,…, j) for each N stress level. The calculation formulas are shown as follows:

$${A:\;U}_{ij}=\frac{X_{ij}-X_{jmin}}{X_{jmax}-X_{jmin}}(Positive\;correlation)$$ (1)

$$B:\;U_{ij}=1-\frac{X_{ij}-X_{jmin}}{X_{jmax}-X_{jmin}}(Negative\;correlation)$$ (2)

Where, Xjmin denotes the minimum value of the adaptation coefficient of N stress for each index of stress level j and Xjmax the maximum value of the adaptation coefficient of N stress for each index of stress level j. The membership function values of all traits under each stress was Ui, and a larger Ui value indicated a stronger ability to adapt to N stress.

Statistical analysis was carried out and means were compared using least significant difference (LSD) test. One-way ANOVA was used with SPSS 26. 0 software and the significance difference in means was analyzed based on the measured values for each trait. To evaluate the effects of the different horizontal nitrogen treatments (sampling time (T), nitrogen treatment (N), and T x N interaction), we analyzed the data using a factorial ANOVA. Data in the graphs are means ± standard deviations (X±S.D.) of three replicates. Mapping was performed using Originlab 8.0.

## 3. Result

### 3.1 Effect of different concentrations of nitrogen treatment on the morphology and chlorophyll content of sugar beet seedlings

**Fig 1:**
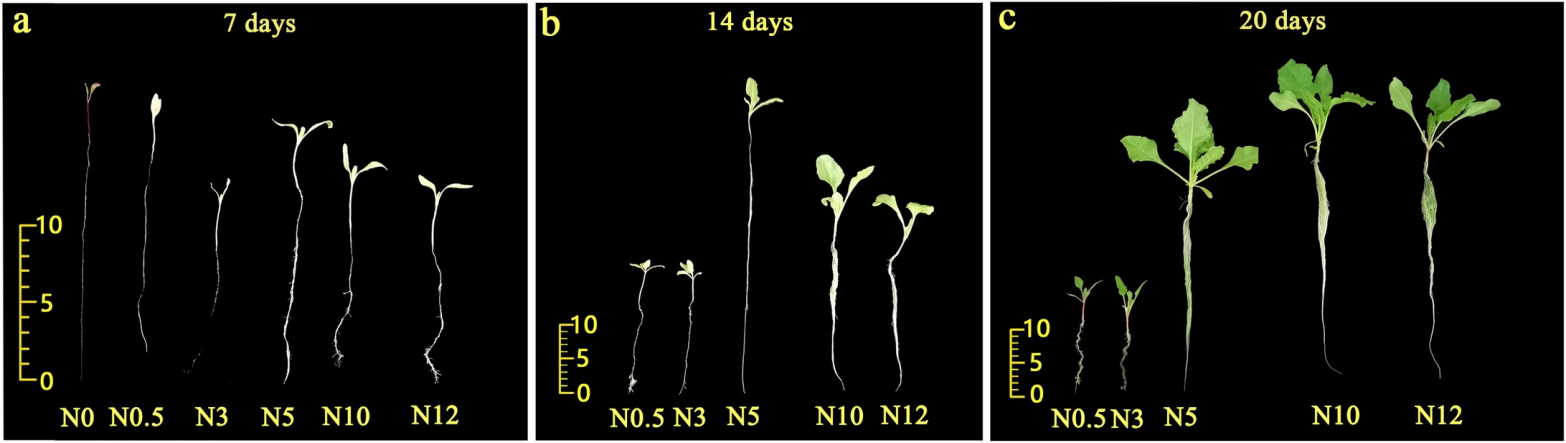
Morphological changes of sugar beet seedlings under different nitrogen treatments.

Significant morphological changes were observed in sugar beet seedlings after 7, 14, and 20 d of N treatments. A standard Hoagland nutrient solution, which provided sufficient N of 5 mmol/L was used as the control. Without N (0 mmol/L), the seedlings could not live and the leaves were yellow and wilted even at 7 d; but the root length increased significantly (Fig 1). Under low N (N0.5 and 3 mmol/L), sugar beet was consistently weaker and smaller as the N concentration decreased, compared with the other treatments. However, high N (N10 and N12 mmol/L) did not influence the growth of seedlings too much, and even after 14 days of stress treatment, the growth potential of the above-ground under high N was better than that of the control. The morphological differences under both low and high N stress were more and more significant with the increasing stress time. Low N was more likely to cause a reduction in the growth of sugar beet seedlings than high N.

**Fig 2:**
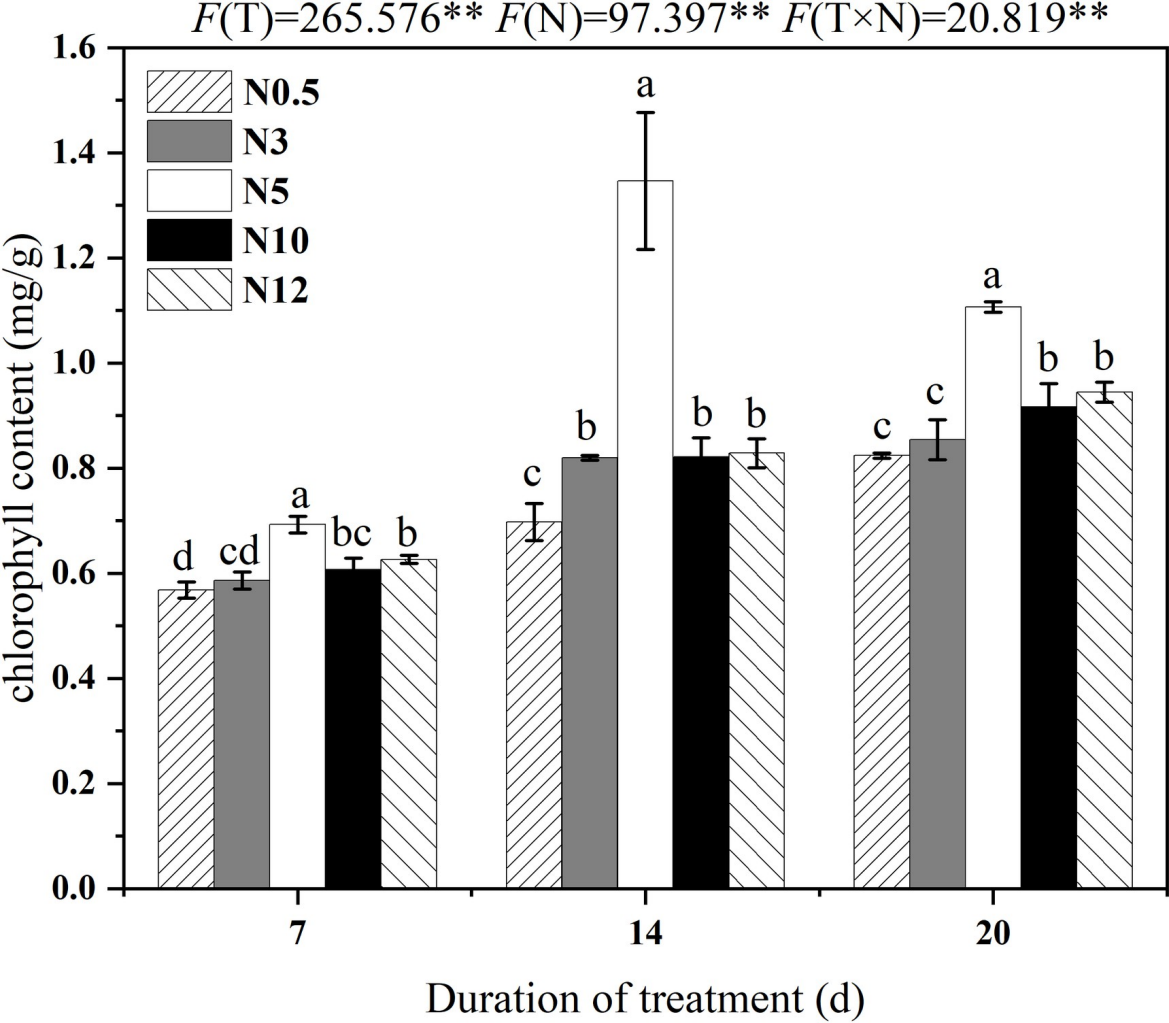
Changes of chlorophyll content in sugar beet seedlings under low and high N stresses. Note: control: N5; low N: N0.5 and N3; high N: N10 and N12; Different letters indicated significant difference among treatments (P<0.05) in the same day, F(T) indicated the F value for duration of treatment, F(N) indicated the F value of N concentration, and F(T×N) denoted F-value for the interaction of stress time and intensity. * and ** indicated significant (P < 0. 05) and (P < 0. 01), respectively. The same below.

Among all the treatments, the highest chlorophyll content was observed when the seedlings were grown under N5 (Fig 2). High N could accumulate more chlorophyll than low N. The short-term stress of N stresses inhibited the production of chlorophyll more severely than that of long-term N stresses. The changes in chlorophyll content might be due to the N adaptation mechanism of sugar beet seedlings with extension of stress time.

### 3.2 Analysis of morphological traits of sugar beet under different nitrogen stresses

As shown in Table 2, there was no significant difference among N treatments on plant height at 7 d; however, N0.5 significantly promoted sugar beet root elongation (by 17.54%) (P < 0.05) compared with the control (N5), and significantly inhibited the growth of leaf surface area (to 68.44%, P < 0.05). At 14 and 20 d, seedlings under N5 exhibited relatively excellent growth performance among all the treatments, while both low and high N treatments significantly inhibited the plant height and root elongation. From these morphological changes, low N restricted the whole growth of sugar beet more than high N.

**Table 2:** Changes in the growth of sugar beet seedlings under different N treatments.

Treatment	Nitrogen stress	Plant height (cm)	Total root length (cm)	Total leaf area (cm^2^/Plant)	Total root surface area (cm^2^/Plant)
7 days	N0.5	3.633±0.551a	15.633±0.814a	2.750±0.101d	22.330±1.979ab
	N3	3.533±0.503a	11.200±0.529b	5.487±1.669c	13.830±7.131bc
	N5	4.533±0.503a	13.300±0.819b	8.713±1.679ab	27.853±10.228a
	N10	4.333±0.379a	12.633±1.662b	10.733±1.082a	11.240±2.126c
	N12	4.100±0.608a	12.033±1.518b	8.207±0.491b	25.657±1.831a
14 days	N0.5	4.400±0.529c	14.500±2.587b	17.103±1.488b	39.790±14.137b
	N3	4.567±0.896c	13.367±2.194b	22.790±2.967ab	60.893±18.965b
	N5	9.900±1.277a	34.667±7.286a	39.003±5.079ab	140.283±84.256a
	N10	8.500±0.520ab	20.400±2.287b	61.960±6.045a	212.573±12.290a
	N12	8.133±0.907b	15.700±4.194b	36.900±22.733ab	152.713±34.151a
20 days	N0.5	5.033±0.723c	13.500±1.375c	55.060±10.000b	321.853±228.267a
	N3	5.900±1.836c	10.767±1.234c	92.437±66.039b	376.830±38.965a
	N5	16.767±1.601a	33.133±3.296a	244.797±82.461a	506.347±140.680a
	N10	16.300±0.500a	34.000±5.292a	196.773±35.786ab	625.690±197.370a
	N12	13.633±1.320b	26.633±4.464b	157.460±119.305ab	459.047±88.552a
Level of significance					
Treatment (T)		235.523**	41.797**	43.974**	91.385**
Nitrogen (N)		80.674**	32.009**	3.654*	4.348**
T×N		23.383**	12.570**	2.994*	1.121

Note: control: N5; low N: N0.5 and N3; high N: N10 and N12; different letters in the same column denote significant differences between different N concentrations in the same day at p< 0.05. F(T) indicated the F value for duration of treatment, F(N) indicated the F value of N concentration, and F(T×N) denoted F-value for the interaction of stress time and intensity.

* and ** indicated significant differences at P < 0.05 and P < 0.01, respectively. The same below.

### 3.3 Influence of different concentrations of nitrogen treatment on the biomass of sugar beet

After 7- and 14-days N treatments, the accumulation of biomass of seedlings was directly proportional to the increase of nitrogen concentration from 0.5 to 10 mmol/L. However, seedlings under N5 showed the largest leaf and root dry weight after 20 days N treatments. In the whole process of high N treatments, N12 restricted the biomass accumulation of sugar beet more than N10. However, root to shoot ratio exhibited different trend, and low N treatment could promote the root elongation (Table 3).

**Table 3:** Effects of different nitrogen treatments on sugar beet biomass.

Treatment	Nitrogen stress	Leaf dry weight (g/Plant)	Root dry weight (g/Plant)	Total dry weight (g/Plant)	Root to shoot ratio
7 days	N0.5	0.004±0.001c	0.002±0.001a	0.006±0.001c	0.639±0.127a
	N3	0.005±0.002bc	0.001±0.000b	0.006±0.002c	0.222±0.096bc
	N5	0.007±0.002ab	0.001±0.001b	0.008±0.002bc	0.150±0.043c
	N10	0.009±0.001a	0.003±0.001a	0.011±0.001a	0.317±0.101b
	N12	0.008±0.001a	0.001±0.001b	0.009±0.002ab	0.126±0.016c
14 days	N0.5	0.012±0.003b	0.004±0.001b	0.016±0.003b	0.378±0.038ab
	N3	0.017±0.004b	0.007±0.002b	0.025±0.006b	0.422±0.068a
	N5	0.060±0.044ab	0.020±0.015ab	0.081±0.059ab	0.336±0.057ab
	N10	0.083±0.031a	0.028±0.011a	0.111±0.042a	0.332±0.004ab
	N12	0.056±0.016ab	0.018±0.008ab	0.073±0.023ab	0.308±0.056b
20 days	N0.5	0.039±0.014c	0.014±0.003c	0.053±0.017c	0.389±0.077a
	N3	0.060±0.023c	0.023±0.011c	0.083±0.034c	0.369±0.070a
	N5	0.532±0.108a	0.163±0.055a	0.695±0.152a	0.307±0.083a
	N10	0.362±0.070b	0.100±0.019b	0.462±0.089b	0.275±0.003a
	N12	0.285±0.051b	0.111±0.025ab	0.396±0.075b	0.387±0.019a
Level of significance					
Treatment (T)		174.079**	91.502**	160.179**	3.960*
Nitrogen (N)		36.007**	16.416**	31.897**	13.399**
T×N		24.974**	11.754**	22.188**	9.070**

### 3.4 Low and high nitrogen stresses restricted nitrogen content in sugar beet seedlings

In general, the N content of the above-ground of sugar beet seedlings was greater than that in the roots. Compared to the control of N5, N content in leaves and roots of sugar beet decreased with the deepening of low N stress, and such phenomenon was more apparent after 20 days treatments. The reduction in total N content of sugar beet seedlings at N0.5 was 64.40%, 33.90%, and 42.63% at 7, 14 and 20 d, respectively. High N supply of N10 and N12 could not enhanced the nitrogen content of seedlings, compared with N5 (Table 4).

**Table 4:** Effects of nitrogen treatments on nitrogen content of sugar beet seedlings.

Treatment	Nitrogen stress	Root N contents (%)	Leaf N contents (%)	Total N contents (%)
7 days	N0.5	1.296±0.163c	1.588±0.082b	2.884±0.148c
	N3	2.476±0.167b	3.423±0.297a	5.899±0.138b
	N5	3.863±0.288a	4.239±0.194a	8.102±0.459a
	N10	3.468±0.310a	3.496±0.962a	6.965±1.257ab
	N12	3.676±0.028a	3.848±0.391a	7.524±0.408a
14 days	N0.5	2.853±0.047d	3.339±0.467c	6.192±0.430d
	N3	3.481±0.187c	3.391±0.313c	6.872±0.481c
	N5	4.628±0.061a	4.741±0.342a	9.369±0.381a
	N10	4.508±0.046a	4.389±0.055ab	8.897±0.015a
	N12	4.279±0.099b	3.963±0.180b	8.242±0.241b
20 days	N0.5	2.380±0.037e	3.049±0.131c	5.429±0.094d
	N3	2.769±0.023d	3.067±0.078c	5.837±0.097c
	N5	4.798±0.021a	4.664±0.067ab	9.463±0.082a
	N10	4.502±0.013c	4.770±0.041a	9.272±0.046b
	N12	4.581±0.035b	4.548±0.121b	9.129±0.155b
Level of significance				
Treatment (T)		19.831**	223.280**	71.700**
Nitrogen (N)		46.819**	445.539**	154.936**
T×N		5.231**	10.835**	7.768**

### 3.5 Changes in protective enzyme activities and malondialdehyde of sugar beet seedlings treated with different concentrations of nitrogen

**Fig 3:**
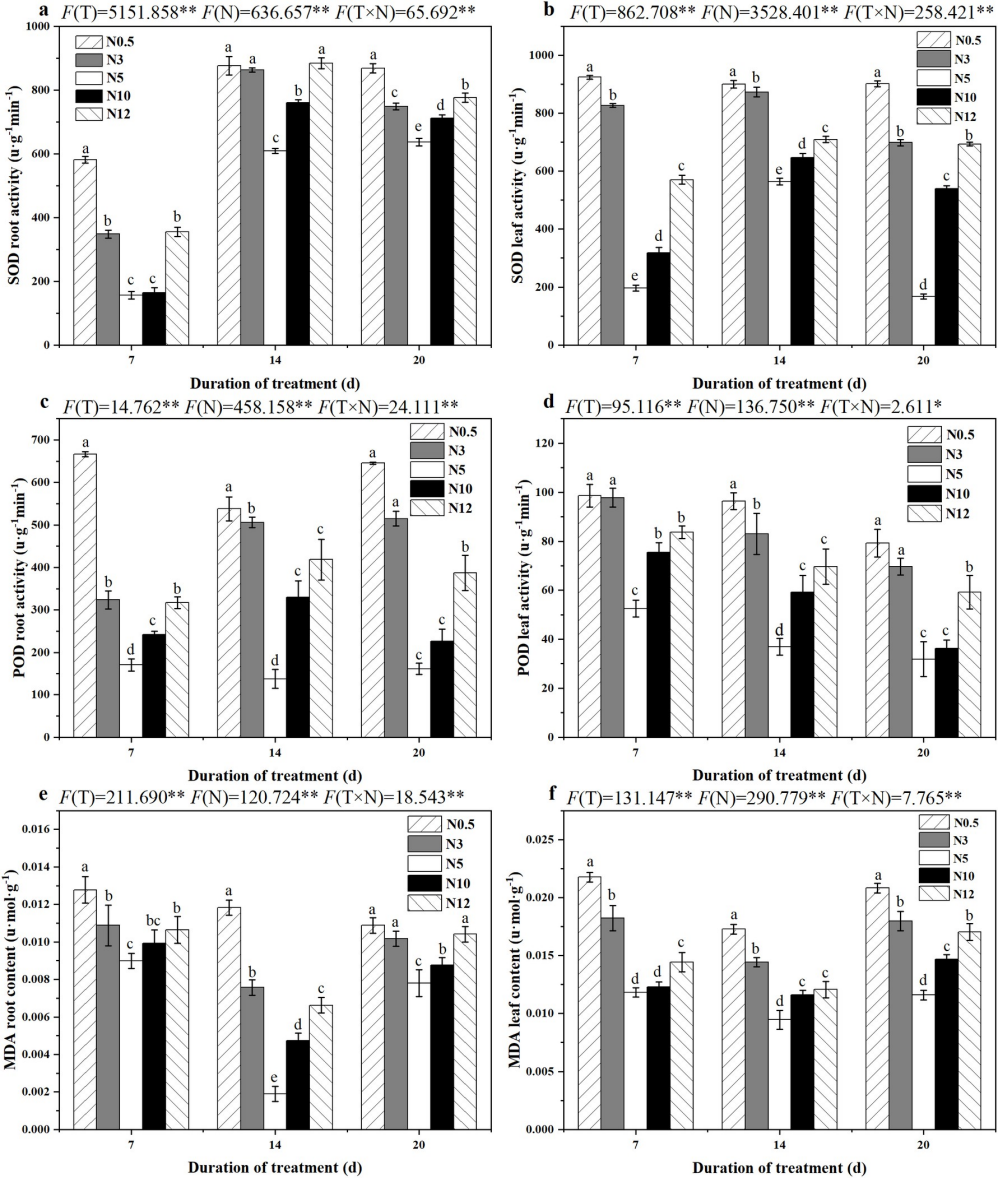
SOD activity (a and b), POD activity (c and d) and MDA content (e and f) in sugar beet seedlings under different nitrogen stresses.

With increased stress time, both low and high N treatments significantly promoted SOD activity in roots and leaves (P < 0.05), compared with N5 (Fig 3A and 3B). All the N stresses could promote POD activity (P < 0.05), which in sugar beet roots was greater than that in leaves, and its activity did not increase or decrease significantly with the duration of each stress treatment (Fig 3C and 3D). However, compared with the control, as the N stress deepened, the production of MDA in leaves and roots increased significantly (P < 0.05), irrespective of low or high N stress (Fig 3E and 3F). On the whole, low N treatment could significantly induce SOD and POD activities and MDA content than high N, especially at N0.5.

### 3.6 Glutamine synthetase, proline and protein in sugar beet seedlings responded to different nitrogen stresses

**Fig 4:**
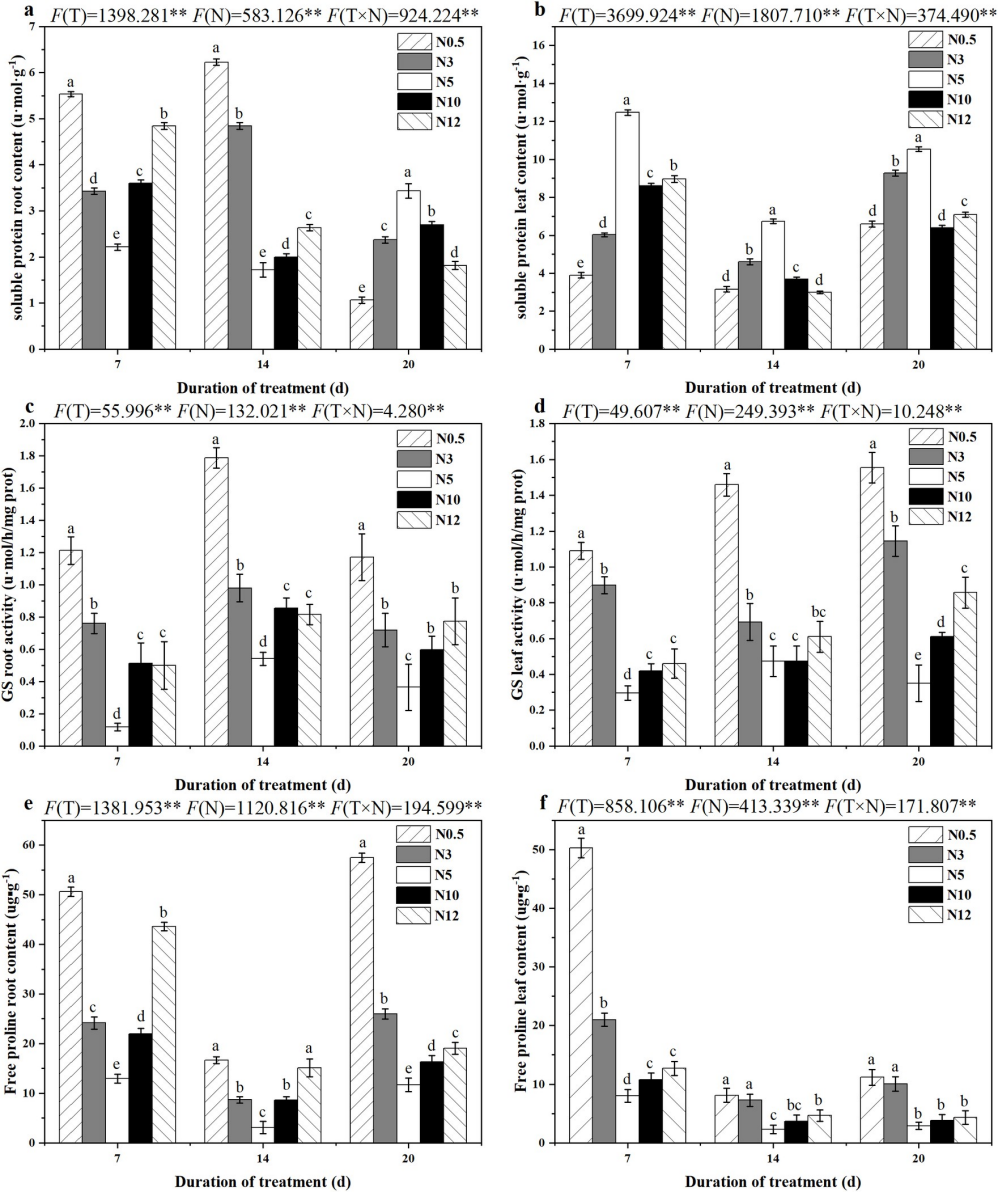
GS activity, protein and proline content of sugar beet seedlings under different nitrogen treatments.

With the aggravation of N stress, the protein content in roots increased first (7 and 14 d) and then decreased (20 d); however, the change in protein content in leaves was opposite. Low N treatments either promoted or inhibited protein accumulation in leaves and roots of sugar beet more significantly than high N treatment (Fig 4A and 4B). Compared with N5, both low and high N treatments could significantly promote GS activity (Fig 4C and 4D) in leaves and roots (P < 0.05), and there was the highest GS activity in seedlings after 14 days of 0.5 mmol/L N treatment. With the N stress time increasing, different from the decreased tendency of the proline content in sugar beet leaves, the proline content in roots increased first and then decreased (Fig 4E and 4F). Compared with N5, 0.5, 3, 10 and 12 mmol/L N promoted proline accumulation significantly (P < 0.05) in roots and leaves of sugar beet, especially N0.5.

### 3.7 Low and high nitrogen stress regulated the transcriptional expression of *BvAMT1*.*2* in sugar beet

**Fig 5:**
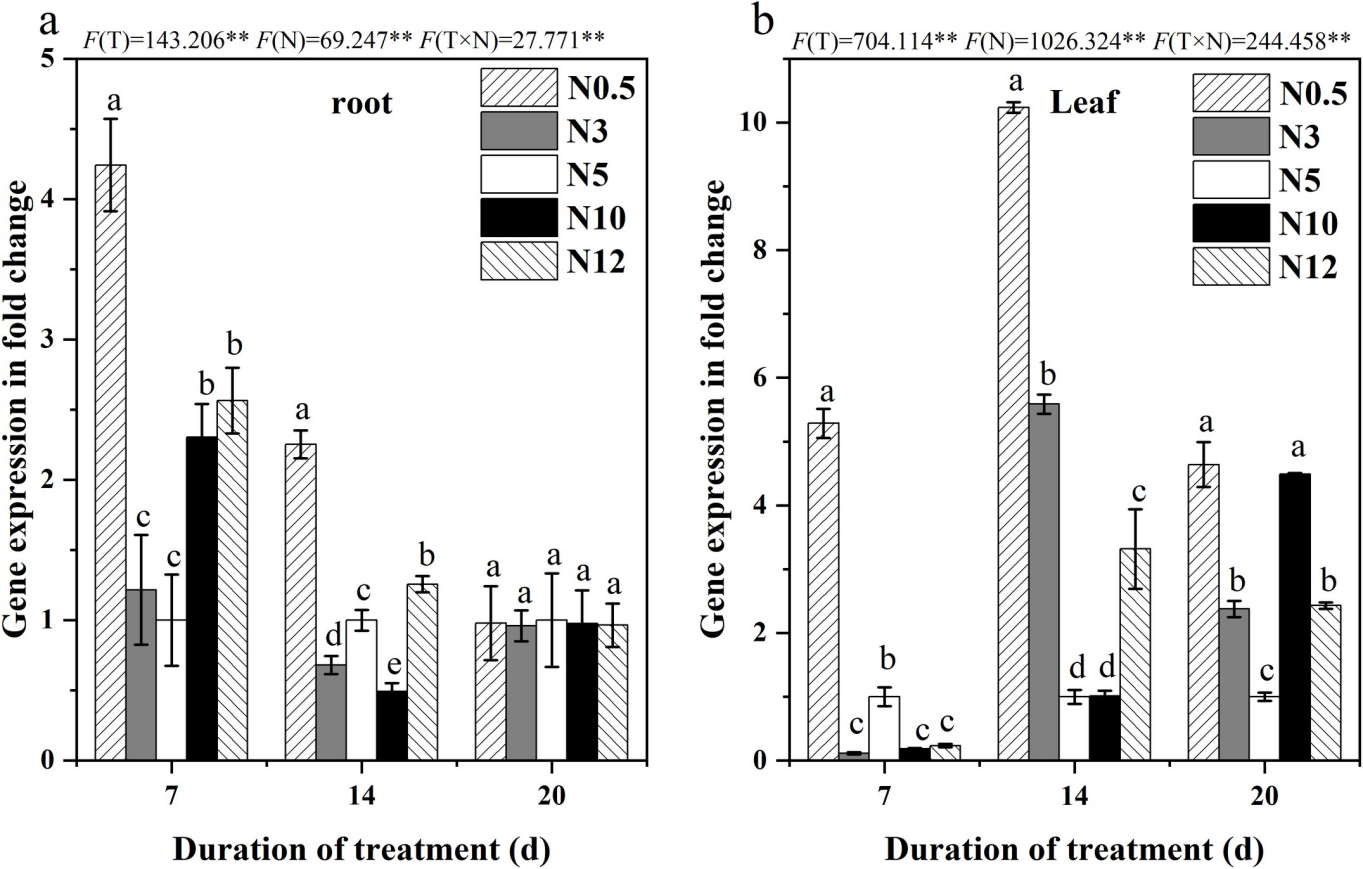
The relative expression pattern of *BvAMT1*.*2* in sugar beet roots (a) and leaves (b) under different nitrogen treatments by qRT-PCR.

The expressional pattern of *BvAMT1*.*2* gene was investigated using qRT-PCR in sugar beet under different N treatments. Compared with the control, *BvAMT1*.*2*’s transcript was induced more significantly by 0.5 mmol/L low N treatment than high N, especially in leaves (Fig 5). There was a maximum of 3.24-or 9.23- fold of *BvAMT1*.*2* up-regulated by N0.5 at 7 d in roots (Fig 5A) or 14 d in leaves (Fig 5B).

### 3.8 Correlation analysis of growth and physiological index and *BvATM1*.*2* expression in sugar beet under different nitrogen treatments

The correlations (r, from—0.995 to 0.999) between the different traits were estimated (Table 5). The total plant N content was significantly positively correlated with plant height (r = 0.980, p < 0.01), and leaf dry weight, root dry weight, and total dry weight (p < 0.05); however, it significantly negatively correlated (p < 0.05) with SOD, POD and MDA. The expression of *BvAMT1*.*2* in leaves exhibited significant positive correlation (p < 0.05) with SOD, POD, GS, proline and MDA in leaves, and MDA and GS in roots; and there was a significant negative correlation (p < 0.05) between the expression of *BvAMT1*.*2* in leaves and plant height, total dry weight and root N content.

**Table 5:** Correlation analysis between different indexes responsive to N stress in sugar beet.

Correlation analysis	Chl	PH	RL	LA	TRSA	LDW	RDW	TDW	RSR	LNC	RNC	TNC	RSOD	LSOD	RPOD	LPOD	MR	ML	SPR	SPL	RGS	LGS	FPR	FPL	AMT1.2 R	AMT1.2 L
Chl	1.000																									
PH	0.722 *	1.000																								
RL	0.951	0.792	1.000																							
LA	-0.399	0.232	-0.129	1.000																						
TRSA	0.285	0.837	0.415	0.618	1.000																					
LDW	0.387	0.889*	0.524	0.583	0.992**	1.000																				
RDW	0.387	0.874	0.521	0.573	0.991**	0.998**	1.000																			
TDW	0.399	0.888*	0.535	0.572	0.990**	0.999**	0.999**	1.000																		
RSR	-0.293	-0.819	-0.415	-0.505	-0.774	-0.791	-0.747	-0.771	1.000																	
LNC	0.643	0.953*	0.672	0.216	0.895*	0.918*	0.919*	0.922*	-0.716	1.000																
RNC	0.781	0.972**	0.875	0.228	0.803	0.869	0.865	0.874	-0.699	0.923*	1.000															
TNC	0.719	0.980**	0.778	0.225	0.870	0.914*	0.912*	0.918*	-0.722	0.985**	0.976**	1.000														
RSOD	-0.913*	-0.749	-0.978**	0.072	-0.443	-0.546	-0.556	-0.563	0.313	-0.668	-0.868	-0.772	1.000													
LSOD	-0.763	-0.992**	-0.837	-0.213	-0.828	-0.885*	-0.878	-0.889*	0.748	-0.954*	-0.993**	-0.990**	0.816	1.000												
RPOD	-0.914*	-0.912*	-0.964**	0.004	-0.626	-0.713	-0.712	-0.722	0.553	-0.844	-0.965**	-0.916*	0.950*	0.947*	1.000											
LPOD	-0.877	-0.941*	-0.903*	-0.008	-0.709	-0.778	-0.781	-0.788	0.579	-0.921*	-0.972**	-0.962**	0.895*	0.970**	0.983**	1.000										
MR	-0.830	-0.889*	-0.817	0.043	-0.717	-0.767	-0.781	-0.781	0.479	-0.935*	-0.919*	-0.946*	0.834	0.925*	0.926*	0.976**	1.000									
ML	-0.786	-0.946	-0.778	-0.024	-0.782	-0.824	-0.827	-0.831	0.628	-0.977**	-0.934*	-0.976**	0.764	0.958*	0.910*	0.970**	0.980**	1.000								
SPR	0.977**	0.632	0.952*	-0.409	0.151	0.266	0.261	0.278	-0.238	0.500	0.708	0.604	-0.904*	-0.674	-0.864	-0.789	-0.706	-0.656	1.000							
SPL	0.923*	0.456	0.843	-0.549	0.055	0.152	0.175	0.175	0.084	0.428	0.578	0.505	-0.861	-0.535	-0.762	-0.717	-0.713	-0.601	0.913*	1.000						
RGS	-0.707	-0.804	-0.617	0.150	-0.681	-0.699	-0.713	-0.710	0.442	-0.915*	-0.778	-0.870	0.619	0.819	0.770	0.871	0.944*	0.949*	-0.540	-0.588	1.000					
LGS	-0.552	-0.753	-0.491	-0.016	-0.767	-0.762	-0.785	-0.774	0.413	-0.911*	-0.735	-0.849	0.531	0.774	0.681	0.803	0.904*	0.904*	-0.364	-0.456	0.970**	1.000				
FPR	-0.820	-0.562	-0.783	0.273	-0.362	-0.429	-0.467	-0.456	-0.013	-0.627	-0.696	-0.671	0.866	0.655	0.796	0.804	0.854	0.735	-0.745	-0.904*	0.744	0.706	1.000			
FPL	-0.768	-0.989**	-0.827	-0.184	-0.829	-0.883*	-0.878	-0.887*	0.729	-0.967**	-0.988**	-0.995	0.809**	0.998**	0.944*	0.976**	0.943*	0.973**	-0.669	-0.547	0.851	0.808	0.674	1.000		
AMT1.2 R	-0.298	-0.442	-0.244	-0.059	-0.621	-0.591	-0.639	-0.612	0.056	-0.680	-0.483	-0.604	0.365	0.497	0.426	0.557	0.716	0.655	-0.111	-0.356	0.787	0.895*	0.692	0.537	1.000	
AMT1.2 L	-0.625	-0.893*	-0.641	-0.183	-0.876	-0.893*	-0.907*	-0.903*	0.586	-0.983**	-0.889*	-0.959	0.670**	0.912*	0.817	0.906*	0.952*	0.964**	-0.468	-0.471	0.939*	0.959*	0.711	0.931*	0.798	1.000

Note: * or ** indicated statistical difference significance at p < 0.05 or p < 0.01 among the treatments by Duncan’s multiple range tests.

Chl: Chlorophyll; PH: Plant Height; RL: Total root length; LA: Total leaf area; TRSA: Total root surface area; LDW: Leaf dry weight; RDW: Root dry weight; TDW: Total dry weight; RSR: Root to shoot ratio; LNC: Leaf N contents; RNC: Root N contents; TNC: Total N contents; RSOD: Root SOD activity; LSOD: Leaf SOD activity; RPOD: POD activity in root; LPOD: POD activity in leaf; MR: MDA in root; ML: MDA in leaf; SPR: Soluble protein in root; SPL: Soluble protein in leaf; RGS: Root GS activity; LGS: Leaf GS activity; FPR: Free proline in root; FPL: Free proline in leaf; AMT1.2 R: Root *BvAMT1*.*2* expression; AMT1.2 L. Leaf *BvAMT1*.*2* expression

### 3.9 Comprehensive evaluation of different nitrogen stresses on sugar beet seedlings

A single indicator was used to evaluate the adaptation mechanism of sugar beet seedlings to high and low N treatments. The growth of seedlings was affected by a combination of multiple factors; therefore, the fuzzy mathematical affiliation function method was used to comprehensively evaluate the 26 indexes, such as chlorophyll, plant height, leaf surface area, root length, root surface area, root to shoot ratio, SOD, POD, and GS activity, of sugar beet seedlings under low and high N treatments (as in Table 6). Based on the mean value of the affiliation function, the ability to adapt to N stress was in the order of: N0.5> N10> N12> N3.

**Table 6:** Comprehensive evaluation of different nitrogen stress ability in sugar beet seedling stage.

Indexes	Nitrogen stress treatments			
	N0.5	N3	N10	N12
Chlorophyll	0.0000	0.1878	0.1917	0.2022
Plant height	0.0000	0.0304	0.7455	0.6787
Total root length	0.0532	0.0000	0.3302	0.1095
Total leaf area	0.4882	0.0000	1.0000	0.4413
Total root surface area	0.0000	0.1221	1.0000	0.6536
Leaf dry weight	0.0001	0.0706	1.0000	0.6198
Root dry weight	0.0004	0.1254	1.0000	0.5835
Total dry weight	0.0001	0.0948	1.0000	0.6000
Root to shoot ratio	0.6141	1.0000	0.2106	0.0001
Leaf N contents	0.0000	0.3538	0.9324	0.8034
Root N contents	0.0000	0.0371	0.7489	0.4451
Total N contents	0.0000	0.2140	0.8513	0.6451
SOD activity in root	0.9720	0.9255	0.5497	1.0000
SOD activity in leaf	1.0000	0.9186	0.2468	0.4326
POD activity in root	1.0000	0.9205	0.4806	0.7024
POD activity in leaf	1.0000	0.7750	0.3750	0.5500
MDA content in root	1.0000	0.5719	0.2864	0.4768
MDA content in leaf	1.0000	0.6368	0.2736	0.3343
Soluble protein in root	0.0001	0.0001	0.0001	0.0001
Soluble protein in leaf	0.0431	0.4292	0.1833	0.0000
GS activity in root	1.0000	0.3517	0.2528	0.2198
GS activity in leaf	1.0000	0.2222	0.0000	0.1389
Free proline in root	1.0000	0.4125	0.4042	0.8875
Free proline in leaf	1.0000	0.8544	0.2427	0.4078
*BvAMT1*.*2* expression in root	1.0000	0.1070	0.0000	0.4340
*BvAMT1*.*2* expression in leaf	1.0000	0.4966	0.0013	0.2507
Membership function mean value	0.5066	0.3792	0.4734	0.4468
Ranking	1	4	2	3

## 4. Discussion

### 4.1 Potential adaptive mechanisms of morphological and biomass response of sugar beet seedlings to different nitrogen stress levels

Nitrogen is a major nutrient that crops absorb from the soil, and it is closely related to crop growth and development. In this study, N supply could also adversely influence sugar beet seedlings, resulted in significant morphological differences under both low and high N stresses, similarly to the findings of Xin [34]. At low N (N0.5 and N3) treatments, beet seedlings exhibited N deficiency traits, including leaf yellowing, plant height and biomass inhibition; under N starvation (N0), the seedlings were almost devoid of normal nutrient supply and could not survive.

Root structure plays an essential function in plant resistance to abiotic stress by sensing outside atmosphere [35]. Previous studies have shown that low N promotes root elongation and facilitated deep root penetration; moderate N increases root number, biomass, and density; while high N inhibits root growth [10, 36, 37]. In this study, under short-term stress, low N inhibited the accumulation of above-ground biomass and root surface area of beet and promoted root length to some extent; while high N stress inhibited leaf production and root surface area, but the effects on biomass did not differ significantly between different N treatments. It seems that the growth of sugar beet under N stress was connected to the duration and concentration of N treatments. This result is similar to the results of Chen et al. [38], where with reduced N, the root system of black cottonwood adjusted in response to stress reaction, leading to enhanced transfer of substances from the fallen leaves to the roots. However, with extended periods of stress, the substances produced by the leaves was not sufficient for root development; therefore, decelerating the root development. As shown in Table 5, plant height and dry weight were significantly positively correlated with N content, and there was significant positive correlation between root surface area and leaf parameters (LDW and LNC). This further suggests that leaf growth depend on the root system for mineral elements, and the growth of the root system needs more nutrients from photosynthesis.

The significant increase in the root to shoot ratio under short-term low N treatment might be due to the change in dry matter accumulated by the sugar beet during growth, even though a certain ratio change existed between above-ground and underground parts at normal growth conditions. Sattelmacher [39] concluded that the growth of crop roots and the above-ground parts is influenced by the balance between carbohydrates and N. From the principle of proximate allocation, the growth of above-ground is limited by N and the root growth by carbon. Thus, N deficiency severely inhibited the accumulation of above-ground biomass, and a large amount of carbon was allocated to the root system to promote root growth and allow the accumulation of underground biomass to capture N. Excessive N application has been shown to delay buckwheat senescence so that carbohydrates and nitrogen could be accumulated in the nutrient organs, but was not a conducive "reservoir" transfer; thus reducing buckwheat yield [40]. Besides, the N content in sugar beet seedlings was highly dependent on the level of N supply, which is consistent with the results of Xin [41].

### 4.2 Regulatory changes in the physiological response and functional gene participation in the tolerance adaptation of sugar beet seedlings to different nitrogen stresses

Plants can adapt to N abnormal not only by efficient N uptaking and utilization, but also through the N allocation to maintain higher chlorophyll content and photosynthetic productivity. Our results showed that both low and high N inhibited chlorophyll synthesis in sugar beet seedlings, especially after 14 days N treatment. This was likely due to the unstable growth of sugar beet seedlings under external stress, disrupting the internal integrity and stability, which in turn influenced photosynthesis [42]. He et al. discovered that N restriction affects photosynthesis by reducing chlorophyll, leading to starch accumulation and causing damage to photosynthetic membranes [43].

In response to adverse stress, plants produce oxygen radicals that induce membrane lipid reactions and generate harmful substances, such as MDA [44], and correspondingly, plant cells evolve antioxidant systems that scavenge ROS to maintain homeostasis [45]. SOD and POD are extremely important enzymes in such antioxidant defense system [46, 47]. Lack or excess of N can promote the development of ROS and cause oxidative stress [48]. With the aggravation of N stress, excessive ROS resulted in cell membrane peroxidation by MDA accumulation, and inhibited of beets’ physiology; however, both low and high N treatments promoted POD and SOD activities, which suggested that these two enzymes participated in anti-oxidation of sugar beet to such stressful conditions. This study also showed that under short-term N stress, the SOD and POD activities in the roots were higher than those in the leaves, and the opposite under long-term stress treatments. These results are similar to the findings of studies on tea trees [47], in which stress effect of plants are transported from the root system to the above-ground. In general, sugar beets can actively create their defense mechanisms to scavenge ROS due to N stress.

It was also found that both low and high N stresses promoted the production of proline sugar beet, where low N promoted higher proline content to regulate the osmotic environment and reduce the damage caused by adversity stress [49]. Biomass accumulation is known to be associated with protein synthesis, and protein synthesis is one of the main targets for controlling growth in response to environmental changes [50]. In this study, we found that both low and high N treatments significantly promoted the production of beet root protein under 7 and 14 d N stress, which possibly induced excess production of stress responsive proteins, consequently increasing plant adaptation ability to stressful conditions [51].

GS could promote the release of ammonia cycle and enhanced plant tolerance to N deficiency under culture conditions [52]. In this study, either low or high N stress promoted GS activity of sugar beet, and GS activity was highly significantly positively correlated with *BvAMT1*.*2* gene expression in both leaves and roots (P < 0.01). *BvAMT1*.*2* was regulated in a N concentration-dependent manner. These results indicated that the *BvAMT1*.*2* gene was more strongly involved in the regulation of N stress in sugar beet by playing a role in the extraction of photorespiratory ammonium—which escapes from the mitochondria—and the entry of xylem ammonium [53].

## 5. Conclusions

**Fig 6:**
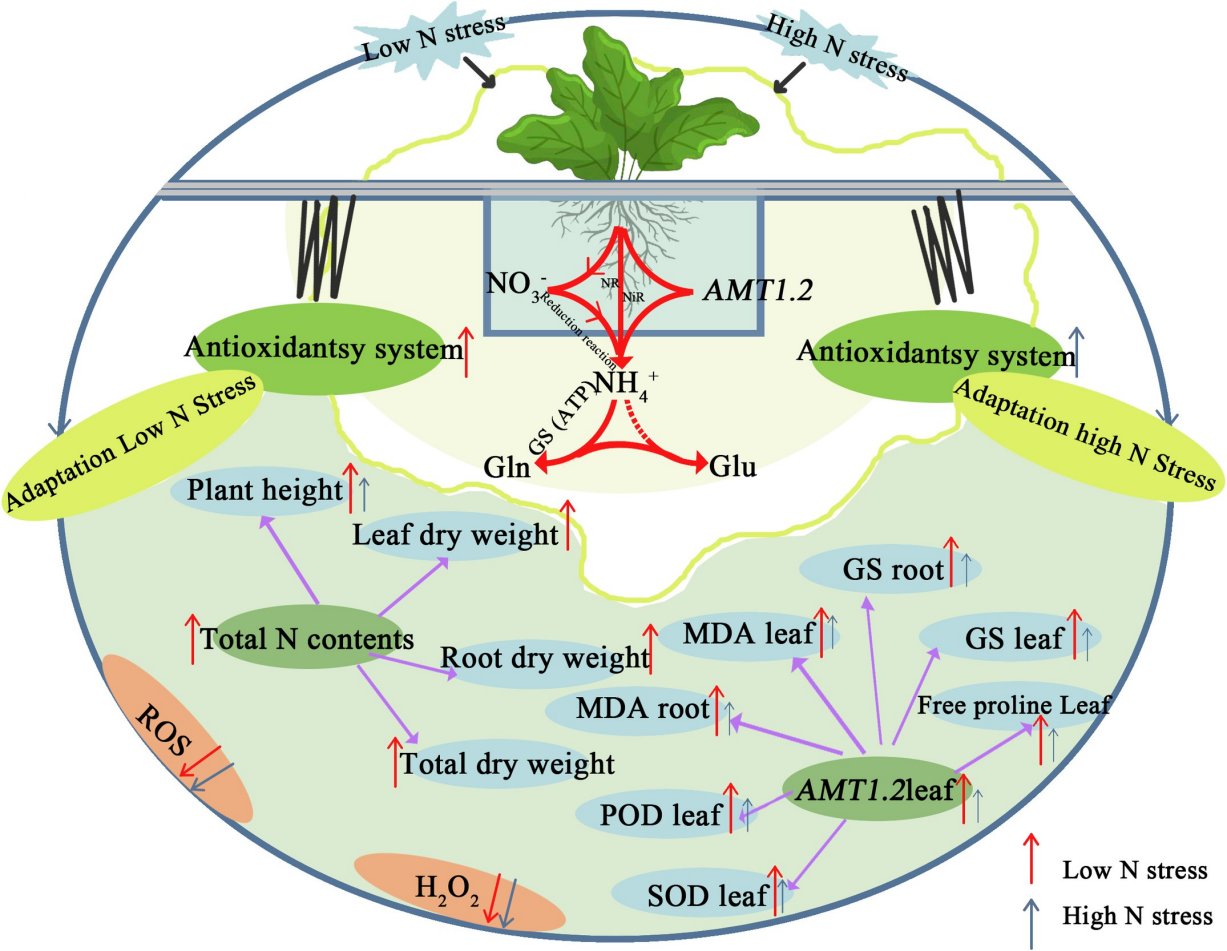
Schematic model of stress growth of sugar beet seedlings under low and high nitrogen conditions.

In all, sugar beet seedlings enable themselves to adapt better to low than high N supply through biological processes such as ROS reduction, protein synthesis, antioxidant enzyme activity regulation, *BvAMT1*.*2* gene expression and assimilating and translocating ammonium, thus conferring the tolerance to maintain optimum growth and development of sugar beet. This study elucidated the responsive mechanism of N-mediated determinants of sugar beet seedling growth, and provided a new idea for improving N use efficiency in sugar beet (Fig 6).

## Supporting information

S1 Table The data of this manuscript. (DOCX)

## Abbreviations

N: Nitrogen
LN: Low Nitrogen
T: Treatment
GS: Glutamine synthetase
ROS: Reactive oxygen species
POD: peroxidase
SOD: Superoxide dismutase
MDA: malondialdehyde
Pro: Proline
TCA: Thiobarbituric acid
PCR: Polymerase chain reaction
qRT-PCR: Quantitative Real-time PCR
AMTs: Ammonia transporter proteins
r: Correlations
Chl: Chlorophyll
PH: Plant Height
RL: Total root length
LA: Total leaf area
TRSA: Total root surface area
LDW: Leaf dry weight
RDW: Root dry weight
TDW: Total dry weight
RSR: Root to shoot ratio
LNC: Leaf N contents
RNC: Root N contents
TNC: Total N contents
RSOD: Root SOD activity
LSOD: Leaf SOD activity
RPOD: POD activity in root
LPOD: POD activity in leaf
MR: MDA in root
ML: MDA in leaf
SPR: Soluble protein in root
SPL: Soluble protein in leaf
RGS: Root GS activity
LGS: Leaf GS activity
FPR: Free proline in root
FPL: Free proline in leaf
AMT1.2 R: Root BvAMT1.2 expression
AMT1.2 L: Leaf BvAMT1.2 expression
