# Supplementary material for: Acclimation of sugar beet in morphological, physiological and BvAMT1.2 expression under low and high nitrogen supply
Source: PLoS One. 2022 Nov 29;17(11):e0278327. doi: 10.1371/journal.pone.0278327 (PMC9707788; doi:10.1371/journal.pone.0278327)
Supplement: S1 Table — (DOCX) [file pone.0278327.s001.docx]

S1 Modified Hoagland nutrient solution formula

| Technical concentration | Solution preparation method | N 0 mmol/L (ml/L) | N 0.5 mmol/L (ml/L) | N 3 mmol/L (ml/L) | N 5 mmol/L (ml/L) | N 10 mmol/L (ml/L) | N 12 mmol/L (ml/L) |
| --- | --- | --- | --- | --- | --- | --- | --- |
| 1 mol/L Ca(NO_3_)_2_·4H_2_O | 236.15 g constant volume 1 L water | - | 0.25 | 1.5 | 2.5 | 5 | 6 |
| 1 mol/L MgSO_4_·7H_2_O | 246.47 g constant volume 1 L water | 2 | 2 | 2 | 2 | 2 | 2 |
| 1 mol/L KH_2_PO_4_ | 136.09 g constant volume 1L water | 1 | 1 | 1 | 1 | 1 | 1 |
| 0.5 mol/L K_2_SO_4_ | 174.26 g constant volume 2 L water | 4.5 | 4.5 | 4.5 | 4.5 | 4.5 | 4.5 |
| 1 mol/L CaCl_2_ | 110.98 g constant volume 1 L water | 6 | 5.75 | 4.5 | 3.5 | 1 | - |
| 0.01 mol/L EDTA-Fe | The table below | 4 | 4 | 4 | 4 | 4 | 4 |
| 0.01 mol/L Mixed micronutrients | The table below | 1 | 1 | 1 | 1 | 1 | 1 |
| EDTA-Fe | weighing（g） | constant volume（L） | | | | | |
| EDTA | 2.9 | 1 | | | | | |
| FeSO_4_∙7H_2_O | 2.8 |  |  |  |  |  |  |
| KOH | 3.3 |  |  |  |  |  |  |
| Mixed trace elements |  |  |  |  |  |  |  |
| H_3_BO_3_ | 8.68 | 3 | | | | | |
| MnSO_4_·H_2_O | 7.5 |  |  |  |  |  |  |
| ZnSO_4_·7H_2_O | 5 |  |  |  |  |  |  |
| CuSO_4_·H_2_O | 0.24 |  |  |  |  |  |  |
| H_2_MoO_4_ | 0.19 |  |  |  |  |  |  |
